# Clinical utility of diagnosing Lynch syndrome in gynecologic oncology: a joint statement from four Japanese academic societies

**DOI:** 10.1007/s10147-026-03126-8

**Published:** 2026-07-11

**Authors:** Shinya Sato, Kenta Masuda, Katsutoshi Oda, Takeshi Kuwata, Takeshi Nakajima, Masayoshi Yamada, Tatsuro Yamaguchi, Akira Hirasawa, Masaki Mandai, Kohji Tanakaya, Hideyuki Ishida, Takayuki Yoshino, Aikou Okamoto

**Affiliations:** 1 Department of Obstetrics and Gynecology, Faculty of Medicine, Tottori University, 36-1 Nishi-Cho, Yonago, Tottori, 683-8504 Japan; 2 Department of Obstetrics and Gynecology, Keio University School of Medicine, 35 Shinanomachi, Shinjyuku-Ku, Tokyo, 160-8582 Japan; 3 Division of Integrative Genomics, Graduate School of Medicine, The University of Tokyo, 7-3-1 Hongo, Bunkyo-Ku, Tokyo, 113-8655 Japan; 4 Department of Genetic Medicine and Services, National Cancer Center Hospital East, 6-5-1 Kashiwanoha, Kashiwa, Chiba, 277-8577 Japan; 5 Division of Hereditary Tumors, Department of Genetic Oncology, Osaka International Cancer Institute, 3-1-69 Otemae, Chuo-Ku, Osaka, 541-8567 Japan; 6 Endoscopy Division, National Cancer Center Hospital, 5-1-1 Tsukiji, Chuo-Ku, Tokyo, 104-0045 Japan; 7 Department of Clinical Genetics, Tokyo Metropolitan Cancer and Infectious Diseases Center Komagome Hospital, 3-18-22 Honkomagome, Bunkyo-Ku, Tokyo, 113-8677 Japan; 8 Department of Clinical Genomic Medicine, Graduate School of Medicine, Dentistry and Pharmaceutical Sciences, Okayama University, 2-5-1 Shikata-Cho, Kita-Ku, Okayama, 700-8558 Japan; 9 Department of Gynecology and Obstetrics, Kyoto University Graduate School of Medicine, 54 Shogoin Kawahara-Cho, Sakyo-Ku, Kyoto, 606-8507 Japan; 10 Department of Surgery, National Hospital Organization Iwakuni Medical Center, 1-1-1 Atago-Machi, Iwakuni, Yamaguchi 740-8510 Japan; 11 Department of Clinical Genetics, Saitama Medical Center, Saitama Medical University, 1981 Kamoda, Kawagoe, Saitama, 350-8550 Japan; 12 Department of Gastroenterology and Gastrointestinal Oncology, National Cancer Center Hospital East, 6-5-1 Kashiwanoha, Kashiwa, Chiba, 277-8577 Japan; 13 Department of Obstetrics and Gynecology, Sanno Hospital, International University of Health and Welfare, 8-10-16 Akasaka, Minato-Ku, Tokyo, 107-0052 Japan

**Keywords:** Lynch syndrome, Endometrial cancer, Ovarian cancer, Mismatch repair, Microsatellite instability, Immune checkpoint inhibitor

## Abstract

Lynch syndrome (LS) is an autosomal dominant cancer predisposition syndrome, associated with substantially increased risks of colorectal and gynecologic malignancies. Endometrial cancer may serve as a sentinel cancer for LS, placing gynecologists and gynecologic oncologists in a key position for early recognition and long-term management. This joint statement was developed through a multidisciplinary process involving the Japan Society of Gynecologic Oncology, the Japan Society of Clinical Oncology, the Japanese Society of Hereditary Tumors, and the Japanese Society for Cancer of the Colon and Rectum. It aims to clarify the clinical utility of diagnosing LS in gynecologic oncology within the Japanese healthcare system. We outline a tumor-first paradigm in which universal mismatch repair immunohistochemistry and/or microsatellite instability testing for endometrial cancer facilitates LS detection and provides actionable biomarkers for systemic therapy. This statement summarizes the clinical impact of LS diagnosis across gynecologic oncology. We highlight patterns of synchronous and metachronous malignancies and the prevalence of LS in ovarian cancer, particularly in endometrioid and clear–cell subtypes. We discuss surgical implications, including risk-reducing hysterectomy with bilateral salpingo-oophorectomy, consideration of concomitant gynecologic risk-reducing surgery during colorectal cancer resection, and individualized ovarian preservation in selected early-stage endometrial cancer or atypical endometrial hyperplasia. We also review the treatment relevance of deficient mismatch repair/microsatellite instability-high status for immune checkpoint inhibitors and emerging fertility-preserving approaches, and address surveillance, cascade testing for relatives, implementation barriers, and the need for multidisciplinary pathways and healthcare system–level support to ensure equitable access to genetic counseling and testing.

## Introduction

Lynch syndrome (LS) is an autosomal dominant cancer predisposition syndrome caused by germline pathogenic variants in *MLH1*, *MSH2*, *MSH6*, or *PMS2*, or by 3′-end deletions of *EPCAM* that silence *MSH2*. LS confers elevated lifetime risks of multiple malignancies, including colorectal, endometrial, and ovarian cancers, as well as cancers of the stomach, small intestine, hepatobiliary tract, pancreas, urinary tract (renal pelvis and ureter), brain, and skin [1, 2]. Although historically identified through families with colorectal cancer, endometrial cancer (EC) frequently precedes colorectal cancer in women and may be the first malignancy within a family, creating an opportunity for early screening or diagnosis and prevention [3]. In women, EC is the most common LS-associated malignancy, and ovarian cancer (OC) risk is also increased, particularly for endometrioid and clear-cell histologies [4]. Consequently, gynecologists and gynecologic oncologists play an important role in LS detection and management.

Beyond risk assessment, LS status can define clinically meaningful subgroups within EC. In the combined PORTEC-1/2/3 trial population, LS prevalence was 2.8% overall and 9.5% among mismatch repair-deficient ECs; while the majority (approximately 70% of the deficient mismatch repair (dMMR) subgroup) are attributed to sporadic *MLH1* silencing by its promoter hypermethylation. LS-associated EC showed a trend toward better recurrence-free survival but a higher risk of second primary cancers compared with *MLH1*-hypermethylated MMR-deficient tumors, underscoring the importance of distinguishing germline-driven disease from sporadic MMR deficiency [5].

The clinical presentation and lifetime cancer risk of LS vary markedly by the underlying germline pathogenic variants. Individuals carrying pathogenic variants in *MLH1* or *MSH2* face high risks for EC (up to 54–57%) and OC (up to 20–38%), with a relatively early median age of presentation (43–49 years). Conversely, while individuals carrying germline pathogenic variants in *MSH6* have a high cumulative risk for EC (up to 49%), they tend to present later, with a median age of 53–55 years. Individuals carrying germline pathogenic variants in *PMS2* exhibit the lowest penetrance; their OC risk (1.3–3.0%) is only slightly higher than that of the general population (1.3%), and the median age of onset is later (49–50 years for EC; 51–59 years for OC) [2, 6]. These gene-specific profiles directly inform genetic counseling on ovarian preservation and the timing of risk-reducing surgery.

**Fig. 1:**
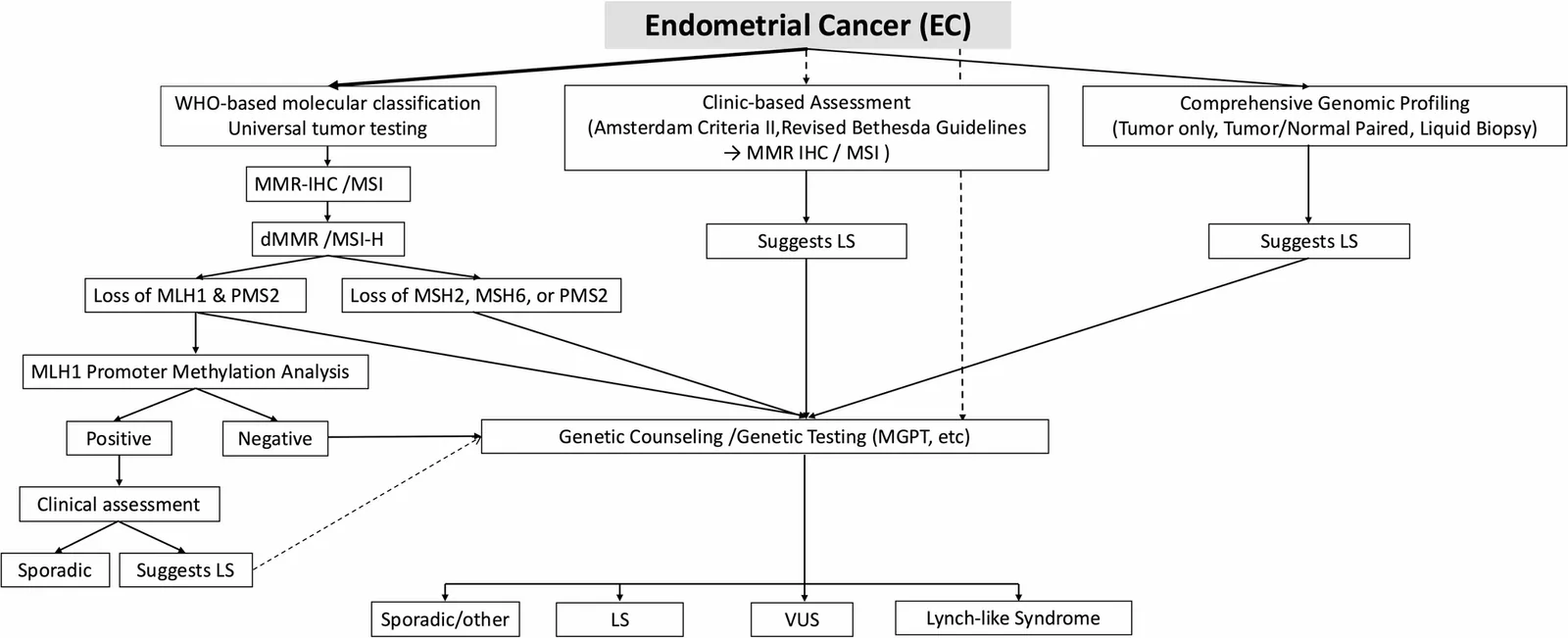
Comprehensive Diagnostic Workflow for Lynch Syndrome in Patients with Endometrial Cancer. The figure illustrates an integrated diagnostic workflow for Lynch syndrome that incorporates WHO-based molecular classification (universal tumor testing), reflex tumor testing, clinic-based assessment, NGS-based comprehensive genomic profiling (CGP), and subsequent germline evaluation. Solid lines: current standard practice in Japan; dashed lines: optional/emerging pathways dependent on institutional resources/coverage. Key Principles and Technical Considerations**:** *Universal Tumor Testing* Systematic screening via MMR-IHC or MSI testing is recommended for all patients to identify potential Lynch syndrome cases. *Genetic Counseling (GC)* Formal GC is a prerequisite for all genetic testing to ensure appropriate informed consent and psychological support. *Established and Emerging Pathways Solid lines* indicate established diagnostic pathways, including universal screening, which has become a standard entry point in current genomic medicine. *Dashed lines* represent personalized or emerging pathways. These include direct referral to GC based on clinical criteria (e.g., Amsterdam Criteria II) and the proactive use of multi-gene panel testing (MGPT) as an evolving option aligned with the latest clinical guidelines (e.g., from Japanese Society of Hereditary Tumors in 2025). *Technical Limitations of NGS***:** When Lynch syndrome is suspected based on tumor testing, definitive germline evaluation using MGPT or other appropriate methods (e.g., MLPA for detecting large deletions/duplications) is essential. EC, endometrial cancer; MMR, mismatch repair; MSI, microsatellite instability; CGP, comprehensive genomic profiling; LS, Lynch syndrome; VUS, variant of uncertain significance

**Fig. 2:**
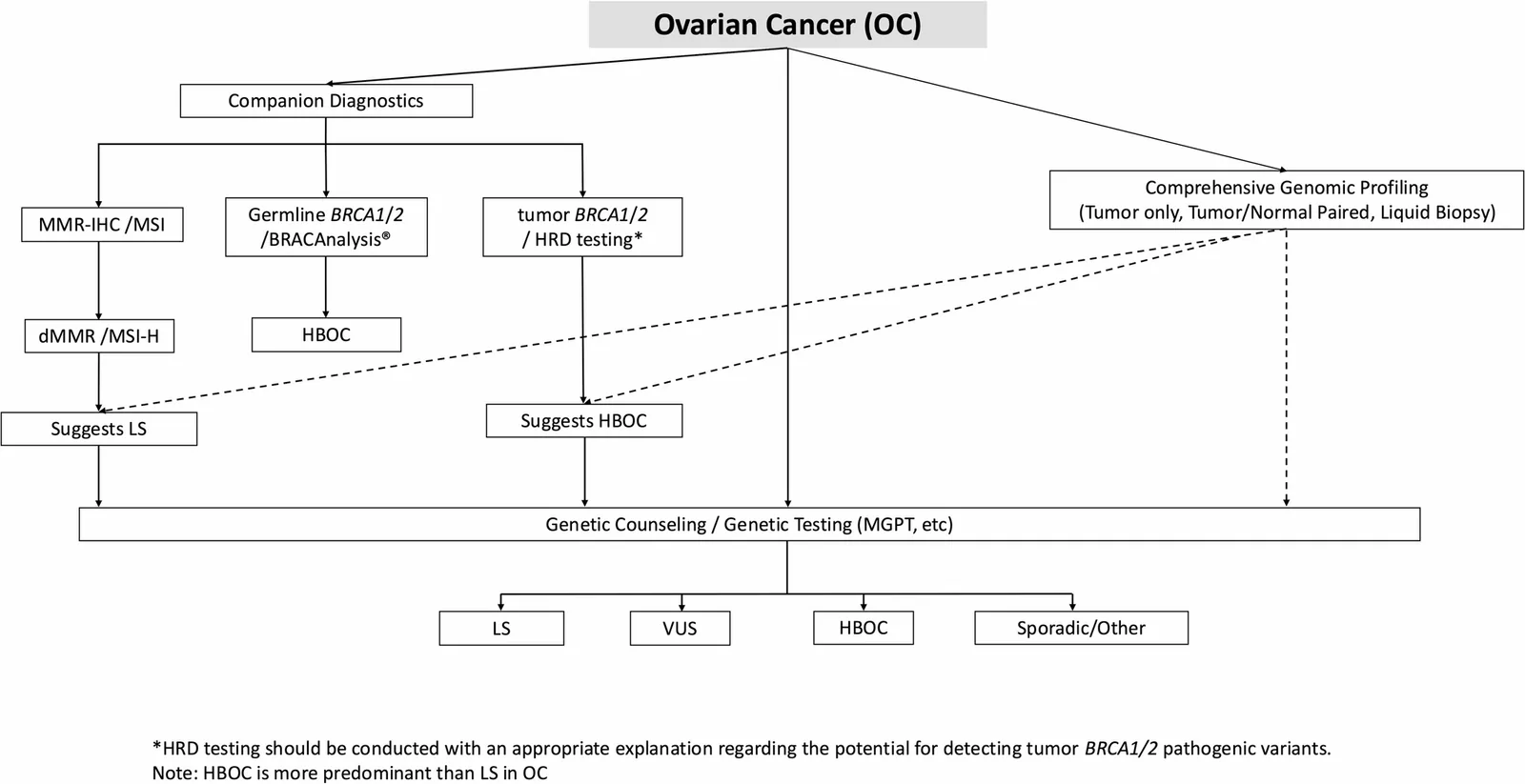
Comprehensive Diagnostic Workflow for Lynch Syndrome and HBOC in Patients with Ovarian Cancer. The figure presents a histology-driven diagnostic strategy for hereditary predispositions, primarily focusing on Lynch syndrome (LS) and Hereditary Breast and Ovarian Cancer (HBOC). *Key Principles and Clinical Considerations* Histology-based Assessment: Patients with non-high-grade serous carcinoma (non-HGSC), such as endometrioid or clear cell carcinoma, should be considered to prioritize for LS screening via MMR-IHC or MSI testing. *HRD and tumor BRCA1/2 Testing*: For patients with HGSC, tumor-based testing (HRD or *BRCA1/2*) is often the primary entry point. These tests should be conducted following an appropriate explanation regarding the potential for detecting germline-implicated variants. *Established and Emerging Pathways* (Solid lines): Direct referral to GC and subsequent germline testing is the standard for cases where HBOC or LS is strongly suspected. *Solid lines* indicate established diagnostic pathways. Dashed lines represent alternative or context-dependent pathways, including CGP-guided referral. *Differential Diagnosis* The workflow emphasizes the importance of formal GC in interpreting complex results, including the distinction between LS, HBOC, and Lynch-like syndrome, particularly when tumor and germline findings are discordant. In the context of ovarian cancer, HBOC is more predominant than LS; however, the possibility of LS should not be overlooked in specific histological subtypes or patients meeting clinical criteria. OC, ovarian cancer; HGSC, high-grade serous carcinoma; MMR, mismatch repair; IHC, immunohistochemistry; MSI, microsatellite instability; HRD, homologous recombination deficiency; HBOC, hereditary breast and ovarian cancer; LS, Lynch syndrome; MGPT, multi-gene panel testing; VUS, variant of uncertain significance

**Fig. 3:**
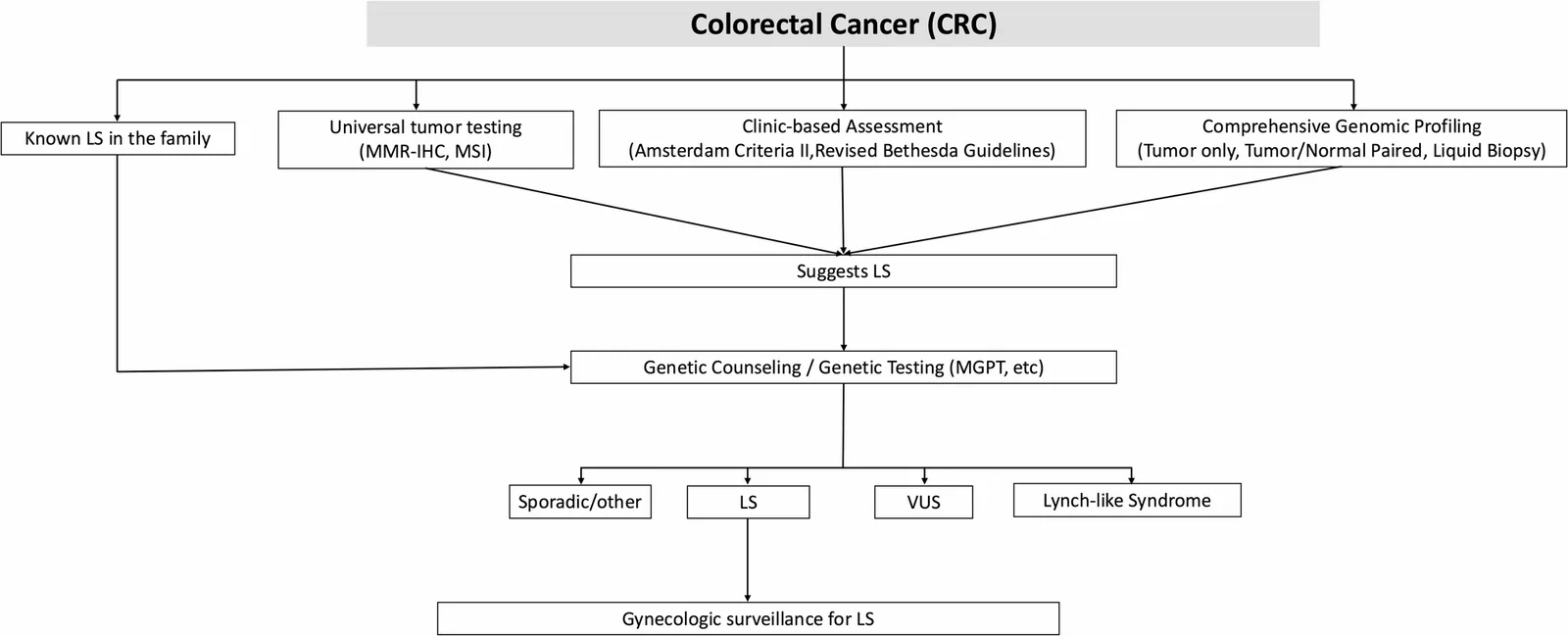
Gynecologic Management Workflow for Women with Colorectal Cancer or a History of Colorectal Cancer in Whom Lynch Syndrome Is Suspected or Confirmed. This figure illustrates a gynecologic management workflow for women with colorectal cancer (CRC), a history of CRC, or a known familial Lynch syndrome (LS)-associated pathogenic variant. *Key Principles and Clinical Considerations* When a pathogenic variant has already been identified in a family member, direct referral to genetic counseling (GC) and subsequent germline testing is recommended. In the absence of a confirmed familial diagnosis, LS should be evaluated through clinical criteria (e.g., Amsterdam Criteria II) and/or tumor-based testing (MMR immunohistochemistry or microsatellite instability testing); if LS is suspected on either basis, referral to GC and germline testing is indicated. Comprehensive genomic profiling (CGP) may also serve as an entry point for identifying LS-associated findings in select cases. Upon confirmation of LS through germline testing, gynecologic surveillance should be discussed, and risk-reducing surgery may be considered in accordance with the patient's reproductive wishes and individual risk profile. MMR, mismatch repair; IHC, immunohistochemistry; MSI, microsatellite instability; LS, Lynch syndrome; MGPT, multi-gene panel testing; VUS, variant of uncertain significance

Over the past decade, diagnostic practice has shifted toward a tumor-first paradigm. Universal tumor testing of newly diagnosed EC for MMR deficiency using immunohistochemistry (IHC) and/or microsatellite instability (MSI) testing identifies more individuals with LS than historical criteria and concurrently provides biomarkers that inform systemic therapy [7–9]. The Japanese Gynecologic Oncology Society (JSGO) formed a Working Group on genomic medicine and hereditary tumors to evaluate optimal LS implementation in gynecologic oncology. In 2025, JSGO developed a statement through multidisciplinary collaboration with the Japan Society of Clinical Oncology (JSCO), the Japanese Society of Hereditary Tumors (JSHT), and the Japanese Society for Cancer of the Colon and Rectum (JSCCR). This four-society statement emphasizes integration of multigene panel testing (MGPT), tumor-based screening, and genetic counseling as part of routine care [10]. The present Special Article is based on that statement and summarizes consensus-based clinical perspectives on the impact of LS diagnosis on management, treatment, prevention, and survivorship in gynecologic oncology. Furthermore, we discuss practical considerations for implementing these strategies in Japanese clinical practice, with particular attention to current limitations in health insurance coverage. Figures 1, 2, 3 summarize an integrated, tumor-first diagnostic workflow for LS that incorporates WHO-based pathology classification and molecular subtyping, together with reflex testing and germline evaluation pathways, including next-generation sequencing (NGS)-based comprehensive genomic profiling (CGP) testing.

## Clinical features relevant to the diagnosis of lynch syndrome

### Frequency of synchronous and metachronous LS-associated cancers

In LS, the development of multiple primary malignancies over a patient’s lifetime is relatively common, particularly involving endometrial, colorectal, and ovarian cancers, either alone or in combination. These malignancies may occur synchronously or metachronously, with important implications for management and long-term surveillance [11, 12].

In the general population, synchronous gynecologic–gastrointestinal or gynecologic double primaries are uncommon. Among 56,986 cases of OC, synchronous EC was observed in fewer than 3% [13]. In a study from an Asian population (Taiwan), the cumulative incidence of synchronous or metachronous double primaries involving endometrial and colorectal cancer among 62,764 women diagnosed with either tumor was only 0.3% [14].

By contrast, synchronous and metachronous cancers occur more frequently in LS. In the Prospective Lynch Syndrome Database (PLSD), among 1942 individuals with LS, 314 developed cancer and 21 (approximately 7%) presented with synchronous malignancies in multiple organs [15]. Moreover, nearly half of women with LS who developed multiple cancers have been reported to have EC or OC as their first malignancy, supporting the concept of gynecologic cancers as a “sentinel cancer” in this setting [12].

Genotype distribution also differs by tumor type and may influence clinical suspicion and downstream testing. In a large multigene-panel cohort of patients with LS who underwent testing for the four MMR genes and *EPCAM*, *MLH1* pathogenic variants were the most frequent among individuals with colorectal cancer only (38.2%), followed by *MSH2/EPCAM* (26.4%), *PMS2* (18.8%), and *MSH6* (16.7%). In contrast, among individuals with OC only or EC only, *MSH6* pathogenic variants were the most frequent (45.0 and 46.5%, respectively), while *MSH2/EPCAM* accounted for 22.5% of OC-only cases and 29.6% of EC-only cases [16]. Of note, the risk of EC may be lower in individuals with isolated 3′-end deletions of *EPCAM* than in those with *MSH2* pathogenic variant carriers [17].

Overall, these findings underscore that synchronous and metachronous gynecologic and gastrointestinal malignancies are characteristic of LS. When a gynecologic cancer is diagnosed first, it may represent the initial (“sentinel”) manifestation and should prompt comprehensive evaluation and long-term surveillance, with particular attention to subsequent gastrointestinal cancer risk.

With respect to LS screening in endometrial or colorectal cancer, many institutions employ a stepwise strategy using MMR-IHC and MSI testing to triage patients for further assessment, as illustrated in Figs. 1 and 3. In selected patients, however, such as those with a known familial LS-associated pathogenic variant or a strong personal or family history suggestive of LS, some experts advocate upfront germline testing at the time of cancer diagnosis, arguing that this may more reliably establish an accurate LS diagnosis [18].

### Prevalence of LS in ovarian cancer (OC)

Most hereditary ovarian cancers are attributable to hereditary breast and ovarian cancer (HBOC) due to germline pathogenic variants in *BRCA1/2*. However, LS is also an important cancer predisposition syndrome in OC, and some cases may remain undiagnosed. This discrepancy between HBOC and LS partly reflects differences in diagnostic pathways and insurance coverage in clinical practice [19]. In OC, germline *BRCA1/2* testing has been widely implemented through BRACAnalysis®, and tumor *BRCA1/2* testing is routine in primary, advanced disease (such as MyChoice CDx ®), enabling systematic identification of both germline and somatic *BRCA1/2* pathogenic variants [20, 21]. In contrast, tumor-based screening for dMMR or MSI is not widely adopted in OC practice. Moreover, even when dMMR or MSI-high (MSI-H) status is identified, confirmatory germline testing for LS is generally not covered by national health insurance in Japan, creating additional barriers to definitive diagnosis.

A systematic review suggests that approximately 9% of ovarian cancers show loss of MMR protein expression by IHC (dMMR) and about 13% demonstrate MSI-H [22]. Importantly, among MSI-H tumors with loss of expression of MLH1, many are attributable to *MLH1* promoter hypermethylation and are not necessarily due to LS. In contrast, MSI-H/dMMR tumors without hypermethylation more often represent LS, and overall, approximately 2% of ovarian cancers are ultimately diagnosed as LS.

The likelihood of MSI-H/dMMR varies by histology: MSI-H is relatively frequent in endometrioid ovarian carcinoma (12%) and in non-serous/non-mucinous subtypes (9%), whereas it is rare in serous carcinoma (1%) [22]. Japanese data are consistent with these observations: 2.6% of 230 OC patients carried pathogenic variants in MMR genes, suggesting that LS may be underrecognized in a subset of ovarian cancers [23]. Overall, LS prevalence in OC is estimated at approximately 2%, but the probability is higher in specific histologic and molecular subtypes, supporting appropriate genetic evaluation in clinical practice. Because penetrance, age at onset, and tumor-testing caveats vary by MMR gene, Table 1 summarizes gene-specific features that directly inform genetic counseling on ovarian preservation, and the timing of risk-reducing surgery, while Table 2 highlights the interpretation of MSI/MMR results.

**Table 1:** Gene-specific features and surgical management for Lynch syndrome

Gene	Colorectal cancer Risk through age 80/Typical age	Endometrial cancer Risk through age 80/Typical age	Ovarian cancer Risk through age 80/Typical age	Risk-reducing surgery timing (counseling)
*MLH1*	46–61%/44 y	34–54%/49 y	4–20%/46 y	Consider total hysterectomy + BSO around age 40 after childbearing; individualize by comorbidities and preferences
*MSH2/EPCAM*	33–52%/44 y	21–57%/47–48 y	8–38%/43 y	Consider total hysterectomy + BSO around age 40 after childbearing (highest OC risk among LS genes)
*MSH6*	10–44%/42–69 y	16–49%/53–55 y	≤ 1–13%/46 y	Consider hysterectomy + bilateral salpingectomy around age 40; discuss delayed oophorectomy (e.g., around age 50) to reduce premature menopause burden
*PMS2*	8.7–20%/61–66 y	13–26%/49–50 y	Insufficient data to confirm elevated risk	Lower penetrance: consider later timing (e.g., around age 50) based on individualized risk–benefit discussion

Risk ranges and typical age of presentation are summarized from NCCN Guidelines v1.2025 (Genetic/Familial High-Risk Assessment: Colorectal, Endometrial, and Gastric)

**Table 2:** Pathological features and tumor testing caveats in lynch syndrome

Gene	Tumor testing notes (MSI/dMMR, concordance)
*MLH1*	MLH1/PMS2 loss often prompts MLH1 promoter methylation testing to distinguish sporadic from LS. In CRC, IHC and MSI show near-perfect concordance; use either as appropriate
*MSH2/EPCAM*	Loss of MSH2/MSH6 strongly suggests LS; proceed to germline *MSH2* ± *EPCAM* deletion testing. IHC and MSI generally concordant
*MSH6*	Isolated MSH6 loss may be MSI-negative (MSS); do not exclude LS based on MSS alone. Prefer MMR-IHC when LS is suspected, and interpret MSI results cautiously
*PMS2*	Isolated PMS2 loss warrants germline testing. Overall penetrance is lower, but LS-related cancers can occur; surveillance decisions should be individualized

Concordance and testing notes reflect evidence in colorectal cancer (population-based concordance), practical guidance for endometrial cancer testing, and reports that isolated MSH6 loss may be MSS by MSI assays

### Role of NGS-based testing in the tumor-first diagnosis for lynch syndrome

With the widespread implementation of comprehensive genomic profiling, MMR gene alterations are increasingly identified through CGP assays, performed for therapeutic decision-making [24, 25]. Although tumor-only sequencing or liquid biopsy cannot reliably distinguish germline from somatic variants, the detection of pathogenic variants in MMR genes should raise suspicion for LS. Tumor–normal paired sequencing allows more direct inference of germline alterations; however, short-read NGS platforms may have limited sensitivity for certain variant types, such as large genomic deletions or duplications, and variants in regions with high sequence homology, notably the *PMS2* gene and its pseudogene *PMS2CL* [26, 27]. Accordingly, confirmatory germline testing should incorporate appropriate complementary methods for copy-number analysis (e.g., multiplex ligation-dependent probe amplification [MLPA]) when clinically indicated [28]. Overall, identification of MMR gene alterations by NGS warrants referral for genetic counseling and consideration of confirmatory germline evaluation, consistent with a tumor-informed, but not tumor-determinative, diagnostic approach (Figs. 1, 2, 3).

Key Practice Points

- Lynch syndrome should be actively considered in patients presenting with multiple primary cancers involving the endometrium, colorectum, and/or ovary. (recommended)
- Lynch syndrome accounts for approximately 2% of ovarian cancers and should not be overlooked, particularly in endometrioid and clear cell carcinomas, in which LS is relatively enriched.

## Impact of the diagnosis of lynch syndrome on surgical management

### Is risk-reducing surgery a viable option for women with LS?

Women with LS have substantially increased lifetime risks of endometrial and ovarian cancers. Although endometrial surveillance (e.g., periodic endometrial biopsy) may be considered, no effective screening strategy has been established for OC [29]. Accordingly, risk-reducing hysterectomy with bilateral salpingo-oophorectomy (BSO) (hereafter, hysterectomy with BSO) is frequently discussed [30]. While prophylactic surgery has not been conclusively shown to reduce mortality, it is highly effective at preventing disease and should be considered, particularly for women who are postmenopausal or have completed childbearing.

Hysterectomy with BSO decreases the incidence of endometrial and ovarian cancers by nearly 100% in cohort data [31]. In addition, for women with LS scheduled for colorectal cancer surgery, concomitant hysterectomy with BSO may be considered to reduce future gynecologic cancer risk [32]. However, PLSD data suggest that uptake at younger ages remains suboptimal: by age 50, 62% of women had undergone hysterectomy and 57% had undergone BSO, whereas concomitant gynecologic risk-reducing surgery at the time of colorectal resection was reported in only 5% [33, 34].

Genetic counseling increasingly incorporates gene- and age-specific considerations in accordance with clinical guidelines when discussing timing and extent of risk-reducing surgery. NCCN v1.2025 summarizes gene-stratified risks and supports individualized genetic counseling: hysterectomy with BSO may be considered around age 40 for *MLH1* and *MSH2/EPCAM* pathogenic variant carriers, whereas for *MSH6* pathogenic variant carriers, a staged approach (e.g., hysterectomy with bilateral salpingectomy around age 40 with consideration of delayed oophorectomy around age 50) may be discussed to balance cancer prevention with consequences of premature menopause; later timing may be appropriate for *PMS2* pathogenic variant carriers given lower penetrance [6, 30] (Table 1).

Accordingly, indications for hysterectomy with BSO should be individualized based on age, genotype, menopausal status, fertility considerations, comorbidities, and patient preferences, using shared decision-making with clear discussion of benefits, limitations (including uncertain mortality impact), and procedure-related consequences [35].

### Ovarian removal vs preservation at hysterectomy in LS patients with stage IA EC (endometrioid carcinoma, G1) or atypical endometrial hyperplasia (AEH)

Reflecting elevated OC risk, several guidelines recommend genetic counseling women with pathogenic variants in *MSH2/EPCAM* regarding hysterectomy with BSO after age 40 [3, 6, 36]. A recent systematic review on cost-effectiveness also supports risk-reducing salpingo-oophorectomy (RRSO) in *MLH1* and *MSH2* pathogenic variant carriers, showing favorable outcomes compared to surveillance alone [37]. NCCN v1.2025 reports cumulative gynecologic cancer risks through age 80 years and age-at-onset distributions by MMR gene, indicating higher EC risk in *MLH1*, *MSH2/EPCAM*, and *MSH6* pathogenic variant carriers (with *MSH6* tending to present later) and the highest OC risk in *MSH2/EPCAM* pathogenic variant carriers, with comparatively lower risk in *MSH6* and *PMS2* pathogenic variant carriers [6].

In contrast, ovarian preservation may be considered in carefully selected young patients with low-risk EC. The 2023 Japanese Guidelines for the Treatment of EC state that, in young patients with endometrioid G1 carcinoma preoperatively estimated as stage IA, ovarian preservation may be proposed if the patient strongly desires it and risks are fully explained [38]. Nevertheless, routine ovarian preservation should be avoided, and in LS, adnexal management requires particularly careful genetic counseling given gene-dependent but consistently increased lifetime OC risk.

Importantly, a definitive LS diagnosis is not always available preoperatively, especially in patients with AEH, in whom universal tumor testing may not be routinely implemented and germline evaluation may not be completed when surgical planning is finalized. From the standpoint of optimal planning, preoperative identification—or at least suspicion—of LS is therefore important not only in EC but also in AEH, because hysterectomy is frequently performed and the choice between ovarian preservation and BSO can substantially affect long-term cancer risk [31].

Key Practice Points

- In women with confirmed LS who have completed childbearing, discuss risk-reducing hysterectomy with BSO as an option through shared decision-making that balances cancer-prevention benefits against menopausal consequences. (recommended)
- Tailor the timing and extent of risk-reducing surgery according to the underlying MMR gene, age, reproductive wishes, menopausal status, and patient preferences, including consideration of staged approaches.
- At the time of colorectal cancer surgery, consider concomitant gynecologic risk-reducing surgery in women with confirmed LS, when appropriate, to reduce cumulative surgical burden.
- In patients with EC/AEH and known or suspected LS, approach ovarian preservation as an exception requiring explicit counseling and careful risk stratification. (recommended)

## Impact of the diagnosis of lynch syndrome on systemic therapy and immunotherapy

The therapeutic landscape for recurrent or advanced gynecologic cancers is increasingly shaped by tumor molecular characteristics, particularly MMR status and MSI. Accordingly, the clinical value of diagnosing LS extends beyond hereditary risk assessment to include biomarker-informed treatment selection and long-term care planning [39].

### Can LS serve as a stratification marker for immune checkpoint inhibitors (ICIs)?

In EC, MMR-IHC and MSI testing are widely used as biomarker testing to guide ICI therapy. A key question is whether MSI-H tumors arising in LS differ in prognosis or ICI sensitivity from MSI-H tumors caused by *MLH1* promoter hypermethylation or from “Lynch-like” tumors. Here, “Lynch-like” tumors refer to cancers that exhibit dMMR or MSI-H status but lack detectable germline pathogenic variants in MMR genes and do not show *MLH1* promoter hypermethylation [40, 41]. Such tumors are thought to arise primarily from biallelic somatic inactivation of MMR genes or from undetected germline variants due to technical limitations (e.g., deep intronic variants or inversions), and represent a biologically distinct subgroup separate from both germline-driven Lynch syndrome and sporadic *MLH1* promoter hypermethylated tumors (Fig. 1 and 3).

Clinically, MSI-H/dMMR endometrial cancers show high responsiveness to PD-1 blockade. Pembrolizumab has been approved for previously treated advanced MSI-H/dMMR endometrial carcinoma based on KEYNOTE-158 and regulatory review [42, 43]. Dostarlimab has also been approved for recurrent or advanced dMMR EC after platinum-based therapy in US and Europe, supported by GARNET and regulatory approval documents [44, 45].

A large study reported that approximately 16% of all MSI-H tumors and 19% of MSI-H endometrial cancers were attributable to LS, and also highlighted that microsatellite stable (MSS) tumors can occasionally arise in LS, particularly among pathogenic variant carriers of lower-penetrance genes such as *MSH6* or *PMS2* [25]. In practice, attenuated MSI signals appear especially relevant to *MSH6*-driven disease [7, 9, 46]. In an analysis of endometrial cancers from clinical trial cohorts, LS tumors classified as MSS by MSI assays frequently showed isolated MSH6 loss on IHC; seven of eight LS tumors categorized as MSS demonstrated isolated MSH6 loss [47]. Biologically, MSH6 functions as part of the MutSα complex (MSH2–MSH6), which recognizes base–base mismatches and small insertion–deletion loops, whereas the alternative MutSβ complex (MSH2–MSH3) preferentially recognizes larger insertion–deletion loops [48]. The precise mechanisms underlying the attenuated MSI phenotype observed in MSH6-deficient tumors remain incompletely understood, but are thought to involve a combination of residual MMR capacity and the inherent sensitivity limitations of standard PCR-based MSI assays [9, 49]. These data, along with the recommendations from the College of American Pathologists (CAP) guidelines [50], support the practical emphasis on MMR-IHC—particularly for identifying MSH6-driven disease—when LS is suspected (Table 2).

Reviews comparing ICI outcomes between LS-associated MSI-H tumors and sporadic MSI-H tumors (often due to *MLH1* hypermethylation) have not consistently demonstrated statistically significant differences in efficacy across colorectal and extracolonic cancers, including EC [51]. Notably, emerging evidence in EC suggests that MSI-H/dMMR tumors caused by *MLH1* promoter hypermethylation may represent a clinically less favorable subgroup compared with LS- or Lynch-like–associated tumors [52, 53]. In a large cohort of 1673 endometrial cancers with *MLH1* aberrations (1492 hypermethylated and 181 mutated), overall survival was significantly worse in *MLH1*-hypermethylated tumors than in LS/Lynch-like cases (hazard ratio = 1.5; median OS, 41.4 vs. 55.9 months; *p* = 0.005) [54]. Consistent with this observation, although based on a small cohort, inferior outcomes following pembrolizumab treatment have been reported in dMMR endometrial cancers with *MLH1* hypermethylation compared with LS/Lynch-like–associated tumors [55]. Taken together, these findings suggest that the underlying mechanism of dMMR—particularly *MLH1* hypermethylation versus germline or Lynch-like alterations—may influence prognosis and possibly ICI responsiveness in EC. Accordingly, when interpreting MSI/MMR results and planning ICI therapy, it may be clinically informative to consider the underlying cause of dMMR (e.g., *MLH1* hypermethylation vs LS/Lynch-like), although whether LS status is an independent predictive biomarker beyond tumor MSI/MMR remains inconclusive [9, 56].

In OC, MSI-H/dMMR is uncommon overall (approximately 1–3%), with exceptional rarity in high-grade serous carcinoma [57]. However, it is enriched in endometrioid and clear-cell histologies [22]. Unlike in EC, where sporadic *MLH1* promoter hypermethylation is the predominant cause of dMMR, dMMR in OC is more frequently attributable to LS, making MMR testing a highly informative screening tool in non-serous carcinomas [58, 59]. Although evidence remains limited compared with EC, the phase II KEYNOTE-158 study demonstrated an objective response rate of 33% (5/15) in patients with MSI-H/dMMR OC, supporting the tissue-agnostic approval of pembrolizumab [60, 61]. MSI-H/dMMR status may help identify a subset of patients who could benefit from immune checkpoint inhibition, consistent with emerging prospective data including dMMR uterine and ovarian cancers [62]. Consequently, current guidelines recommend universal MMR/MSI testing for endometrioid, clear-cell, and mucinous ovarian cancers to identify both individuals with LS and candidates for immunotherapy [63]. For LS patients with recurrent OC, enrollment in clinical trials is therefore particularly important.

### Is fertility-sparing treatment feasible in LS-associated endometrial cancer (EC)?

For selected young women with EC, fertility-sparing hormonal therapy using medroxyprogesterone acetate (MPA) may be considered [38]. However, LS-associated MSI-H EC may exhibit reduced sensitivity to conventional progestin-based therapy, and success rates may be limited in this population [64].

Recently, single-agent ICIs, which are primarily indicated for advanced or recurrent MSI-H EC, have been highlighted as an alternative fertility-sparing strategy in selected cases. Reported cases of young women with LS-associated MSI-H EC resistant to hormonal therapy achieved uterine preservation after ICI treatment, with tumor regression enabling fertility preservation [65]. A case report also described a patient with LS and synchronous endometrial and colorectal cancers who received ICI therapy, preserved uterus and adnexa, and subsequently achieved childbirth [66]. Translating this to EC, the ongoing phase IIb SATELLITE study is currently evaluating dostarlimab for early-stage dMMR EC to assess if similar organ-preserving efficacy can be achieved [67].

Although current evidence is limited to case reports, these observations suggest that ICIs may represent a future fertility-preserving option for LS-associated EC, particularly for tumors refractory to MPA. In clinical practice, discussions with patients should address oncologic safety, expected treatment duration and monitoring, contingency plans for non-response or recurrence (including timely salvage surgery), and referral for genetic counseling and cascade testing.

Key Practice Points

- Use tumor dMMR/MSI-H status to guide ICI eligibility. (recommended)
- When available, interpret the underlying cause of dMMR—such as LS, Lynch-like alterations, or sporadic *MLH1* promoter hypermethylation—as clinically informative, although its independent predictive value remains unproven.
- In ovarian cancer, consider ICI therapy for MSI-H/dMMR disease and prioritize clinical trial enrollment whenever feasible, given the rarity of this subgroup and the limited evidence base. (recommended)
- For selected patients with dMMR/MSI-H endometrial cancer, present fertility-sparing ICI approaches as investigational and based on limited evidence, with clear contingency plans for non-response or recurrence.

## Surveillance, prevention, and familial implications of lynch syndrome diagnosis

After an LS diagnosis, intensified surveillance is recommended to enable early detection of metachronous malignancies. For colorectal cancer, colonoscopy every 1–2 years beginning at age 20–25 years (or earlier depending on family history) is widely recommended [68]. Regarding chemoprevention, long-term data from the CAPP2 randomized controlled trial demonstrated that daily aspirin significantly reduces the risk of colorectal cancer in individuals with LS, establishing it as a preventive option to be discussed with patients [69]. For EC, symptom education and consideration of periodic endometrial sampling starting at age 30–35 years have been proposed, although the survival benefit of screening remains uncertain [68, 70]. For OC, no screening strategy has demonstrated a clear survival benefit; nevertheless, some clinicians consider CA-125 testing and transvaginal ultrasound in selected high-risk individuals, recognizing the limitations of this approach [70]. For urinary tract cancers, which occur more frequently in *MSH2* pathogenic variant carriers, some guidelines suggest consideration of periodic urinalysis or imaging-based surveillance, although evidence for survival benefit remains limited [2, 6].

A key benefit of LS diagnosis is identification of at-risk relatives. Cascade genetic testing enables surveillance and preventive strategies in pathogenic variant carriers and represents a major pathway by which LS diagnosis yields benefit beyond the index patient. Despite growing consensus on the diagnostic approach for LS, implementation barriers remain, including limited access to genetic counseling, variability in institutional resources for tumor testing and MGPT, and patient anxiety or misunderstanding of genetic information. Streamlined consent processes, clinician education, and coordinated multidisciplinary pathways may improve uptake and equity of care.

Key Practice Points

- Implement regular colonoscopic surveillance as a core component of long-term LS care, and discuss chemoprevention options when appropriate. (recommended)
- Emphasize symptom education and individualized prevention strategies for gynecologic cancers, recognizing that the survival benefit of gynecologic screening —particularly ovarian cancer screening—remains unproven. (recommended)
- Prioritize cascade testing of at-risk relatives, supported by genetic counseling, to extend preventive benefit beyond the index patient. (recommended)
- Address Japan-specific barriers to LS care, including limited access to genetic counseling, institutional variability, and out-of-pocket costs, through streamlined referral pathways and multidisciplinary coordination.

## Other considerations

In LS, metachronous development of related malignancies—particularly endometrial, ovarian, and colorectal cancers—may necessitate repeated pelvic and/or abdominal interventions over a patient’s lifetime [71, 72]. Subsequent procedures (e.g., retroperitoneal lymphadenectomy or reoperation) may be more technically challenging because of prior surgery, altered anatomy, and adhesions, potentially increasing postoperative complications including ileus. Therefore, treatment strategies should be individualized, incorporating cancer history, anticipated future cancer risk, and cumulative surgical burden when planning initial and subsequent interventions. These considerations further underscore the clinical value of timely LS diagnosis for optimizing long-term care planning and shared decision-making across the disease trajectory.

## Applicability to Japanese clinical practice and implementation challenges

Much of the evidence informing LS management—including gene-specific penetrance estimates, age-at-onset distributions, uptake of risk-reducing surgery, and outcomes of surveillance—derives from North American and European cohorts such as the PLSD and related studies; recent European consensus recommendations have further summarized prevention and risk-reduction strategies for women with LS [1, 2, 73]. While these datasets provide an indispensable foundation, several factors warrant careful interpretation when translating them into Japanese clinical practice (Table 3).

**Table 3:** Consensus-based practice considerations for Lynch syndrome diagnosis and management in Japanese gynecologic oncology practice

Clinical domain	Consensus-based practice point	Evidence basis	Japanese implementation issue
Endometrial cancer testing	Universal MMR-IHC and/or MSI testing should be considered for newly diagnosed endometrial cancer	International guidelines/observational evidence	Facility-level variability
MLH1 loss	*MLH1* promoter hypermethylation testing is useful to distinguish sporadic MLH1-silenced tumors from cases requiring further evaluation for LS	Tumor-first diagnostic evidence	*MLH1* promoter hypermethylation testing is not reimbursed under the Japanese health insurance system
Ovarian cancer	dMMR/MSI-H ovarian carcinoma, especially endometrioid/clear-cell carcinoma, warrants referral for genetic counseling and consideration of germline evaluation	Limited but consistent evidence	Genetic testing for LS is not covered by Japanese health insurance system
Risk-reducing Surgery	Hysterectomy with BSO may be discussed as an option after childbearing, particularly for carriers of pathogenic variants in *MLH1* or *MSH2*/*EPCAM*	Cohort data/guideline-based	Prophylactic Surgery for LS is not covered by Japanese health insurance system
Ovarian preservation for endometrial cancer/atypical endometrial hyperplasia	May be considered only in highly selected cases	Expert consensus/limited evidence	Requires careful genetic counseling
Fertility-preserving ICI therapy	Investigational option in selected dMMR/MSI-H endometrial cancer	Case-level/early evidence	Prefer clinical trials or expert centers
Cascade testing	Should be offered to relatives	Strong preventive rationale	Genetic testing for LS is not covered by Japanese health insurance system
National Registry/Database	Priority variables should include gene-specific prevalence/age, OC histotype-specific LS frequency, risk-reducing surgery uptake, colonoscopic surveillance quality/outcomes, cascade testing rates, and real-world ICI outcomes	Evidence gap	Need for coordinated national LS registry development
Genetic counseling /genetic testing	MMR-IHC/MSI results or clinical features suggestive of LS should trigger genetic counseling and germline evaluation. (see Figs. 1, 2, 3)	International guidelines	Genetic counseling and genetic testing are not covered for LS in Japanese health insurance system

First, gene-specific penetrance and age at onset may vary across populations, and Japanese epidemiologic data remain limited. Available Japanese studies suggest potential under-recognition of LS in gynecologic oncology and highlight system-level barriers that may influence ascertainment, including variable adoption of tumor testing and limited access to confirmatory germline testing after identification of tumor dMMR/MSI-H [19–23]. Consequently, penetrance estimates and uptake rates reported in Western cohorts should be viewed as informative but not definitive for Japanese patients, and local data collection is a priority.

Second, the histologic distribution of LS-associated ovarian cancer and the clinical pathways used for hereditary cancer testing differ in Japan, where germline *BRCA1*/*2* testing and tumor *BRCA*/HRD testing are widely implemented for ovarian cancer, whereas routine MMR/MSI testing and subsequent germline evaluation for LS are less consistently available [19–21]. This structural imbalance can systematically bias recognition of LS compared with HBOC, and may lead to missed opportunities for cascade testing and prevention among relatives.

Third, access and implementation constraints within the Japanese healthcare system substantially affect the real-world feasibility of international recommendations. Even when tumor-first screening identifies dMMR/MSI-H, barriers to genetic counseling capacity, institutional variability in testing infrastructure, and out-of-pocket costs for germline testing and counseling—especially for unaffected relatives—can limit completion of the diagnostic pathway and downstream prevention [10, 19]. Cost-effectiveness analyses based on non-Japanese healthcare systems may therefore overestimate the practicality of recommended strategies unless implementation barriers are explicitly addressed [37].

Fourth, systemic therapy implications, particularly the use of ICIs in dMMR/MSI-H tumors, are increasingly relevant across tumor types; however, generalizability of efficacy, toxicity profiles, and treatment access should be interpreted within Japanese regulatory and reimbursement contexts and with awareness of genotype- and tumor-type–dependent MSI/MMR phenotypes [6, 22, 39]. In addition, fertility-sparing use of ICIs remains an emerging concept supported mainly by case reports and early-phase studies; its application in Japan should be framed as investigational and pursued preferably within clinical trials [65–67].

To improve Japanese-specific evidence and equitable implementation, a national-level registry/database is warranted. A pragmatic approach would integrate: (i) tumor-based screening results (MMR-IHC/MSI, *MLH1* methylation), (ii) germline and somatic findings (including paired tumor–normal testing where available), (iii) cancer histology and other relevant clinicopathologic characteristics, (iv) uptake and timing of risk-reducing surgery and surveillance, (v) treatment outcomes, including ICI use, and (vi) cascade testing outcomes in relatives. Experience from the Prospective Lynch Syndrome Database (PLSD) further suggests that surveillance outcomes should be evaluated not only by cancer incidence and age at diagnosis, but also by surveillance-related variables and, where feasible, quality indicators [74]; this is particularly relevant when assessing the real-world effectiveness of colonoscopic surveillance in Japan.

Such a framework—aligned with the multidisciplinary pathways described in this statement—would enable more accurate Japanese penetrance estimates, identify gaps in care pathways, and inform policy decisions regarding reimbursement and resource allocation [10].

## Conclusion

Early diagnosis of LS meaningfully influences clinical management in gynecologic oncology by enabling individualized surgical decision-making, biomarker-informed systemic treatment strategies, tailored surveillance, and familial risk assessment. Consideration of concomitant hysterectomy with BSO at the time of colorectal cancer surgery and careful genetic counseling regarding ovarian preservation in EC and AEH are key clinical implications. Coordinated multidisciplinary pathways involving gynecology, colorectal surgery, pathology, and genetics services are essential and may improve outcomes and quality of life. In Japan, out-of-pocket costs for germline testing, genetic counseling, and surveillance remain barriers for both affected individuals and at-risk relatives. In particular, confirmatory germline testing for LS after tumor-based screening may require out-of-pocket payment even in affected patients, and preventive interventions such as surveillance and risk-reducing surgery for unaffected LS carriers are not consistently supported within the current health insurance framework. Further optimization of the healthcare system, including appropriate insurance coverage for these confirmatory and preventive interventions, would improve access to personalized medicine. Establishing a national registry that links tumor-based screening, germline/somatic findings, uptake of preventive interventions and surveillance, and clinical outcomes would provide robust Japan-specific epidemiologic data and support evidence-based policy decisions.

## References

[1] Win AK, Lindor NM, Young JP et al (2012) Risks of primary extracolonic cancers following colorectal cancer in Lynch syndrome. J Natl Cancer Inst 104(18):1363–1372 22933731 10.1093/jnci/djs351 PMC3529597

[2] Dominguez-Valentin M, Sampson JR, Seppälä TT et al (2020) Cancer risks by gene, age, and gender in 6350 carriers of pathogenic mismatch repair variants: findings from the Prospective Lynch Syndrome Database. Genet Med 22(1):15–25 31337882 10.1038/s41436-019-0596-9 PMC7371626

[3] Tanakaya K, Yamaguchi T, Hirata K et al (2026) Japanese Society for Cancer of the Colon and Rectum (JSCCR) guidelines 2024 for the clinical practice of hereditary colorectal cancer. Int J Clin Oncol 31(1):1–66 41214301 10.1007/s10147-025-02892-1 PMC12770061

[4] Bounous VE, Robba E, Perotto S et al (2022) Gynecological cancers in Lynch syndrome: a comparison of the histological features with sporadic cases of the general population. J Clin Med 11(13):3689 35806973 10.3390/jcm11133689 PMC9267402

[5] Post CCB, Stelloo E, Smit VTHBM et al (2021) Prevalence and prognosis of Lynch syndrome and sporadic mismatch repair deficiency in endometrial cancer. JNCI J Natl Cancer Inst 113(9):1212–1220 33693762 10.1093/jnci/djab029 PMC8418420

[6] National Comprehensive Cancer Network. NCCN Clinical Practice Guidelines in Oncology (NCCN Guidelines®): Genetic/Familial High-Risk Assessment: Colorectal, Endometrial, and Gastric. Version 1.2025.

[7] Hampel H, Frankel W, Panescu J et al (2006) Screening for Lynch syndrome (hereditary nonpolyposis colorectal cancer) among endometrial cancer patients. Cancer Res 66(15):7810–7817 16885385 10.1158/0008-5472.CAN-06-1114

[8] Leenen CH, van Lier MG, van Doorn HC et al (2012) Prospective evaluation of molecular screening for Lynch syndrome in patients with endometrial cancer ≤ 70 years. Gynecol Oncol 125(2):414–420 22306203 10.1016/j.ygyno.2012.01.049

[9] Stelloo E, Jansen AML, Osse EM et al (2017) Practical guidance for mismatch repair-deficiency testing in endometrial cancer. Ann Oncol 28(1):96–102 27742654 10.1093/annonc/mdw542

[10] Opinions and Guidelines “The impact of the diagnosis of Lynch syndrome in gynecologic practice on treatment strategy or clinical utility.” Available from: https://jsgo.or.jp/opinion/pdf/lynch-views-guidance.pdf [in Japanese]. Accessed 2026/06/01.

[11] Soliman PT, Slomovitz BM, Broaddus RR et al (2004) Synchronous primary cancers of the endometrium and ovary: a single institution review of 84 cases. Gynecol Oncol 94(2):456–462 15297188 10.1016/j.ygyno.2004.05.006

[12] Lu KH, Dinh M, Kohlmann W et al (2005) Gynecologic cancer as a “sentinel cancer” for women with hereditary nonpolyposis colorectal cancer syndrome. Obstet Gynecol 105(3):569–574 15738026 10.1097/01.AOG.0000154885.44002.ae

[13] Bandera EV, Williams MG, Demissie K et al (2009) Synchronous primary ovarian and endometrial cancers: a population-based assessment of survival. Obstet Gynecol 113(4):783–789 19305320 10.1097/AOG.0b013e31819c7bdf

[14] Chao A, Wu RC, Chao AS et al (2022) Synchronous/metachronous endometrial and colorectal malignancies in Taiwanese women: a population-based nationwide study. Arch Gynecol Obstet 306(1):165–172 35001183 10.1007/s00404-021-06296-0

[15] Møller P, Seppälä T, Bernstein I et al (2017) Cancer incidence and survival in Lynch syndrome patients receiving colonoscopic and gynaecological surveillance: first report from the Prospective Lynch Syndrome Database. Gut 66(3):464–472 26657901 10.1136/gutjnl-2015-309675 PMC5534760

[16] Espenschied CR, LaDuca H, Li S et al (2017) Multigene panel testing provides a new perspective on Lynch syndrome. J Clin Oncol 35:2568–2575 28514183 10.1200/JCO.2016.71.9260 PMC7186580

[17] Kempers MJE, Kuiper RP, Ockeloen CW et al (2011) Risk of colorectal and endometrial cancers in EPCAM deletion-positive Lynch syndrome: a cohort study. Lancet Oncol 12(1):49–55 21145788 10.1016/S1470-2045(10)70265-5 PMC3670774

[18] Chao AS, Chao A, Lai CH et al (2024) Comparison of immediate germline sequencing and multi-step screening for Lynch syndrome detection in high-risk endometrial and colorectal cancer patients. J Gynecol Oncol 35(1):e1 37743058 10.3802/jgo.2024.35.e5 PMC10792205

[19] Yokoyama T, Yamamoto Y, Okazawa-Sakai M et al (2025) Current management of hereditary cancer syndromes in ovarian and endometrial cancer: a Japanese study. Jpn J Clin Oncol. 10.1093/jjco/hyaf176 41215483 10.1093/jjco/hyaf176

[20] Enomoto T, Aoki D, Hattori K et al (2019) The first Japanese nationwide multicenter study of BRCA mutation testing in ovarian cancer: CHARacterizing the cross-sectionaL approach to Ovarian cancer geneTic TEsting of BRCA (CHARLOTTE). Int J Gynecol Cancer 29(6):1043–1049 31263023 10.1136/ijgc-2019-000384

[21] Oda K, Aoki D, Tsuda H et al (2023) Japanese nationwide observational multicenter study of tumor BRCA1/2 variant testing in advanced ovarian cancer. Cancer Sci 114(1):271–280 36254756 10.1111/cas.15518 PMC9807512

[22] Mitric C, Salman L, Abrahamyan L et al (2023) Mismatch repair deficiency, microsatellite instability, and Lynch syndrome in ovarian cancer: a systematic review and meta-analysis. Gynecol Oncol 170:133–142 36682091 10.1016/j.ygyno.2022.12.008

[23] Hirasawa A, Imoto I, Naruto T et al (2017) Prevalence of pathogenic germline variants detected by multigene sequencing in unselected Japanese patients with ovarian cancer. Oncotarget 8(68):112258–112267 29348823 10.18632/oncotarget.22733 PMC5762508

[24] Kawamura M, Shirota H, Niihori T et al (2023) Management of patients with presumed germline pathogenic variant from tumor-only genomic sequencing: a retrospective analysis at a single facility. J Hum Genet 68(6):399–408 36804482 10.1038/s10038-023-01133-5

[25] Habano E, Ogawa M, Watanabe K et al (2026) Germline pathogenic variants detected by GenMineTOP: insight from a nationwide tumor/normal paired comprehensive genomic profiling test, in Japan. J Hum Genet 71(1):1–11 40926019 10.1038/s10038-025-01389-z PMC12689426

[26] Latham A, Srinivasan P, Kemel Y et al (2019) Microsatellite instability is associated with the presence of Lynch syndrome pan-cancer. J Clin Oncol 37(4):286–295 30376427 10.1200/JCO.18.00283 PMC6553803

[27] Vaughn CP, Hart KJ, Samowitz WS et al (2011) Avoidance of pseudogene interference in the detection of 3’ deletions in PMS2. Hum Mutat 32(9):1063–1071 21618646 10.1002/humu.21540

[28] Rako I, Vinceljak E, Popovic M et al (2025) The application of the NGS and MLPA methods in the molecular diagnostics of Lynch syndrome. Diagnostics (Basel) 15(23):2950 41374331 10.3390/diagnostics15232950 PMC12690997

[29] Lindor NM, McMaster ML, Lindor CJ et al (2008) Concise handbook of familial cancer susceptibility syndromes—second edition. J Natl Cancer Inst Monogr 38:1–93 10.1093/jncimonographs/lgn001 18559331

[30] Crosbie EJ, Ryan NAJ, Arends MJ et al (2019) the manchester international consensus group recommendations for the management of gynecological cancers in lynch syndrome. Genet Med 21(10):2390–2400 30918358 10.1038/s41436-019-0489-y PMC6774998

[31] Schmeler KM, Lynch HT, Chen LM et al (2006) Prophylactic surgery to reduce the risk of gynecologic cancers in the Lynch syndrome. N Engl J Med 354(3):261–269 16421367 10.1056/NEJMoa052627

[32] Vasen HFA, Blanco I, Aktan-Collan K et al (2013) Revised guidelines for the clinical management of Lynch syndrome (HNPCC): recommendations by a group of European experts. Gut 62(6):812–823 23408351 10.1136/gutjnl-2012-304356 PMC3647358

[33] Seppälä TT, Dominguez-Valentin M, Crosbie EJ et al (2021) Uptake of hysterectomy and bilateral salpingo-oophorectomy in carriers of pathogenic mismatch repair variants: a Prospective Lynch Syndrome Database report. Eur J Cancer 148:124–133 33743481 10.1016/j.ejca.2021.02.022 PMC8916840

[34] Seppälä TT, Dominguez-Valentin M, Sampson JR et al (2021) Prospective observational data informs understanding and future management of Lynch syndrome: insights from the Prospective Lynch Syndrome Database (PLSD). Fam Cancer 20(1):35–39 32507935 10.1007/s10689-020-00193-2 PMC7870755

[35] Dominguez-Valentin M, Crosbie EJ, Engel C et al (2021) Risk-reducing hysterectomy and bilateral salpingo-oophorectomy in female heterozygotes of pathogenic mismatch repair variants: a Prospective Lynch Syndrome Database report. Genet Med 23(4):705–712 33257847 10.1038/s41436-020-01029-1 PMC8026395

[36] The Japanese Society of Hereditary Tumors (JSHT) (2025) Guidance for multi-gene panel testing (MGPT) for hereditary cancer syndromes. Kanehara & Co., Ltd.

[37] Wei X, Oxley S, Sideris M et al (2022) Cost-effectiveness of risk-reducing surgery for breast and ovarian cancer prevention: a systematic review. Cancers (Basel) 14(24):6117 36551605 10.3390/cancers14246117 PMC9776851

[38] Japan Society of Gynecologic Oncology (JSGO) (2023) Guidelines for treatment of uterine body neoplasms 2023 edition. Kanehara & Co., Ltd.

[39] Concin N, Matias-Guiu X, Cibula D et al (2025) ESGO–ESTRO–ESP guidelines for the management of patients with endometrial carcinoma: update 2025. Lancet Oncol 26:e423–e435 40744042 10.1016/S1470-2045(25)00167-6

[40] Lefol C, Sohier E, Baudet C et al (2021) Acquired somatic MMR deficiency is a major cause of MSI tumor in patients suspected for “Lynch-like syndrome” including young patients. Eur J Hum Genet 29(3):482–488 33279946 10.1038/s41431-020-00778-6 PMC7940425

[41] Ito T, Yamaguchi T, Kumamoto K et al (2024) Incidence and molecular characteristics of deficient mismatch repair conditions across nine different tumors and identification of germline variants involved in Lynch-like syndrome. Int J Clin Oncol 29(7):953–963 38615286 10.1007/s10147-024-02518-y PMC11196295

[42] O’Malley DM, Bariani GM, Cassier PA et al (2022) Pembrolizumab in patients with microsatellite instability-high advanced endometrial cancer: results from the KEYNOTE-158 study. J Clin Oncol 40(7):752–761 34990208 10.1200/JCO.21.01874 PMC8887941

[43] U.S. Food and Drug Administration. FDA approves pembrolizumab for advanced endometrial carcinoma. 2022.

[44] Oaknin A, Tinker AV, Gilbert L et al (2022) Clinical activity and safety of the anti-programmed death 1 monoclonal antibody dostarlimab for patients with recurrent or advanced mismatch repair-deficient endometrial cancer: a nonrandomized phase 1 clinical trial. J Immunother Cancer 10(2):e003777 33001143 10.1001/jamaoncol.2020.4515 PMC7530821

[45] U.S. Food and Drug Administration. FDA grants accelerated approval to dostarlimab-gxly for dMMR endometrial cancer. 2021.

[46] Umar A, Boland CR, Terdiman JP et al (2004) Revised Bethesda Guidelines for hereditary nonpolyposis colorectal cancer (Lynch syndrome) and microsatellite instability. J Natl Cancer Inst 96(4):261–268 14970275 10.1093/jnci/djh034 PMC2933058

[47] Sowter P, Gallon R, Hayes C et al (2024) Detection of mismatch repair deficiency in endometrial cancer: assessment of IHC, fragment length analysis, and amplicon sequencing based MSI testing. Cancers (Basel) 16:3970 39682157 10.3390/cancers16233970 PMC11640224

[48] Jiricny J (2006) The multifaceted mismatch-repair system. Nat Rev Mol Cell Biol 7(5):335–346 16612326 10.1038/nrm1907

[49] Hampel H, Frankel WL, Martin E et al (2005) Screening for the Lynch syndrome (hereditary nonpolyposis colorectal cancer). N Engl J Med 352(18):1851–1860 15872200 10.1056/NEJMoa043146

[50] Vikas P, Messersmith H, Compton C et al (2023) Mismatch repair and microsatellite instability testing for immune checkpoint inhibitor therapy: ASCO endorsement of College of American Pathologists guideline. J Clin Oncol 41(10):1943–1948 36603179 10.1200/JCO.22.02462

[51] Therkildsen C, Jensen LH, Rasmussen M et al (2021) An update on immune checkpoint therapy for the treatment of Lynch syndrome. Clin Exp Gastroenterol 14:181–197 34079322 10.2147/CEG.S278054 PMC8163581

[52] Ozawa R, Nishikawa T, Yoshida H et al (2024) Unveiling pembrolizumab effectiveness in diverse subtypes of MSI-high endometrial cancers. J Gynecol Oncol 35(6):e89 38725237 10.3802/jgo.2024.35.e103 PMC11543255

[53] Kitamura S, Taguchi A, Tamai K et al (2025) DNA methylation in ovarian and endometrial cancers: predictive and mechanistic roles in PARP inhibitor and ICI response. Cancer Sci 116(11):2929–2939 40938267 10.1111/cas.70189 PMC12580874

[54] Toboni MD, Wu S, Farrell A et al (2023) Differential outcomes and immune checkpoint inhibitor response among endometrial cancer patients with *MLH1* hypermethylation versus *MLH1* “Lynch-like” mismatch repair gene mutation. Gynecol Oncol 177:132–141 37683549 10.1016/j.ygyno.2023.08.015

[55] Bellone S, Roque DM, Siegel ER et al (2022) A phase 2 evaluation of pembrolizumab for recurrent Lynch-like versus sporadic endometrial cancers with microsatellite instability. Cancer 128(6):1206–1218 34875107 10.1002/cncr.34025 PMC9465822

[56] Loughrey MB, McGrath J, Coleman HG et al (2021) Identifying mismatch repair-deficient colon cancer: near-perfect concordance between immunohistochemistry and microsatellite instability testing in a large, population-based series. Histopathology 78(3):401–413 32791559 10.1111/his.14233

[57] Xi Q, Kage H, Ogawa M et al (2023) Genomic landscape of endometrial, ovarian, and cervical cancers in japan from the database in the center for cancer genomics and advanced therapeutics. Cancers (Basel) 16(1):136 38201563 10.3390/cancers16010136 PMC10778092

[58] Rambau PF, Duggan MA, Ghatage P et al (2016) Significant frequency of *MSH2*/*MSH6* abnormality in ovarian endometrioid carcinoma supports histotype-specific Lynch syndrome screening in ovarian carcinomas. Histopathology 69(2):288–297 26799366 10.1111/his.12934

[59] Atwal A, Snowsill T, Dandy MC et al (2022) The prevalence of mismatch repair deficiency in ovarian cancer: a systematic review and meta-analysis. Int J Cancer 151(9):1626–1639 35792468 10.1002/ijc.34165 PMC9539584

[60] Maio M, Ascierto PA, Manzyuk L et al (2022) Pembrolizumab in microsatellite instability high or mismatch repair deficient cancers: updated analysis from the phase II KEYNOTE-158 study. Ann Oncol 33(9):929–938 35680043 10.1016/j.annonc.2022.05.519

[61] U.S. Food and Drug Administration. FDA grants accelerated approval to pembrolizumab for first tissue/site-agnostic indication. 2017. Available at: https://www.fda.gov/drugs/resources-information-approved-drugs/fda-grants-accelerated-approval-pembrolizumab-first-tissuesite-agnostic-indication. Accessed 2026/06/01.

[62] Friedman CF, Snyder A, Tao Y et al (2024) Nivolumab for mismatch-repair-deficient or hypermutated uterine or ovarian cancers: a phase 2 study. Nat Med 30:2753–2763 10.1038/s41591-024-02942-7 PMC11108776 38653864

[63] Konstantinopoulos PA, Norquist B, Lacchetti C et al (2020) Germline and somatic tumor testing in epithelial ovarian cancer: ASCO guideline. J Clin Oncol 38(11):1222–1245 31986064 10.1200/JCO.19.02960 PMC8842911

[64] Dagher C, Manning-Geist B, Ellenson LH et al (2023) Molecular subtyping in endometrial cancer: a promising strategy to guide fertility preservation. Gynecol Oncol 179:180–187 37992549 10.1016/j.ygyno.2023.11.006 PMC10843754

[65] Yang X, Xue Y, Shao W et al (2025) Fertility-sparing treatment outcomes using immune checkpoint inhibitors in endometrial cancer patients with Lynch syndrome. J Gynecol Oncol 36(1):e11 39853260 10.3802/jgo.2025.36.e59 PMC12226331

[66] Cao D, Gao Y, Zhang RX et al (2022) Case report: reproductive organ preservation and subsequent pregnancy for an infertility patient with Lynch syndrome-associated synchronous endometrial cancer and colon cancer after treatment with a PD-1 checkpoint inhibitor. Front Immunol 13:1010490 36325347 10.3389/fimmu.2022.1010490 PMC9618861

[67] Obermair A, Gebski V, Goh J et al (2025) Phase 2b, open-label, single-arm, multicenter pilot study of the efficacy, safety, and tolerability of dostarlimab in women with early-stage mismatch repair-deficient endometrioid endometrial adenocarcinoma. Int J Gynecol Cancer 35(4):101644 39955187 10.1016/j.ijgc.2025.101644

[68] Giardiello FM, Allen JI, Axilbund JE et al (2014) Guidelines on genetic evaluation and management of Lynch syndrome: a consensus statement by the U.S. Multi-Society Task Force on Colorectal Cancer. Gastrointest Endosc 80(2):197–220. 10.1016/j.gie.2014.06.006 25034835 10.1016/j.gie.2014.06.006

[69] Burn J, Sheth H, Elliott F et al (2020) Cancer prevention with aspirin in hereditary colorectal cancer (Lynch syndrome), 10-year follow-up and registry-based 20-year data in the CAPP2 study: a double-blind, randomized, placebo-controlled trial. Lancet 395(10240):1855–1863 32534647 10.1016/S0140-6736(20)30366-4 PMC7294238

[70] Idos G, Valle L. Lynch syndrome. In: Adam MP, Bick S, Mirzaa GM, et al., eds. GeneReviews®. University of Washington, Seattle; 2021. Available at: https://www.ncbi.nlm.nih.gov/books/NBK1211/. Accessed 2026/06/01. 20301390

[71] Win AK, Lindor NM, Winship I et al (2013) Risks of colorectal and other cancers after endometrial cancer for women with Lynch syndrome. J Natl Cancer Inst 105(4):274–279 23385444 10.1093/jnci/djs525 PMC3576323

[72] Aung YK, Zhang Y, Jenkins MA, et al (2025) Risks of colorectal and extracolonic cancers following colorectal cancer: a systematic review and meta-analysis. JNCI Cancer Spectr. 9(3):pkaf031. 10.1093/jncics/pkaf031 PMC12086680 40115972

[73] Marchetti C, Gultekin M, Sassu CM et al (2026) ESGO consensus statement on endometrial cancer prevention, risk reduction strategies, and management of women with Lynch syndrome. Eur J Cancer 240:116739 42090914 10.1016/j.ejca.2026.116739

[74] Seppälä TT, Ahadova A, Dominguez-Valentin M et al (2019) Lack of association between screening interval and cancer stage in Lynch syndrome may be accounted for by over-diagnosis; a prospective Lynch syndrome database report.Hered Cancer Clin Pract 28(17):8 10.1186/s13053-019-0106-8 PMC6394091 30858900

